# Novel variants in *DNAH9* lead to nonsyndromic severe asthenozoospermia

**DOI:** 10.1186/s12958-021-00709-0

**Published:** 2021-02-20

**Authors:** Dongdong Tang, Yanwei Sha, Yang Gao, Jingjing Zhang, Huiru Cheng, Junqiang Zhang, Xiaoqing Ni, Chao Wang, Chuan Xu, Hao Geng, Xiaojin He, Yunxia Cao

**Affiliations:** 1 Reproductive Medicine Center, Department of Obstetrics and Gynecology, the First Affiliated Hospital of Anhui Medical University, No 218 Jixi Road, Hefei, 230022 Anhui China; 2 NHC Key Laboratory of study on abnormal gametes and reproductive tract (Anhui Medical University), No 81 Meishan Road, Hefei, 230032 Anhui China; 3 Key Laboratory of Population Health Across Life Cycle (Anhui Medical University), Ministry of Education of the People’s Republic of China, No 81 Meishan Road, Hefei, 230032 Anhui China; 4 Department of Andrology, United Diagnostic and Research Center for Clinical Genetics, School of Public Health & Women and Children’s Hospital, Xiamen University, Xiamen, 361005 Fujian China; 5 State Key Laboratory of Molecular Vaccinology and Molecular Diagnostics & Center for Molecular Imaging and Translational Medicine, School of Public Health, Xiamen University, Xiamen, 361102 China

**Keywords:** Asthenozoospermia, Nonsyndromic, Flagellum, *DNAH9*, ICSI

## Abstract

**Background:**

Asthenozoospermia is one of the most common causes of male infertility, and its genetic etiology is poorly understood. DNAH9 is a core component of outer dynein arms in cilia and flagellum. It was reported that variants of *DNAH9* (OMIM: 603330) might cause primary ciliary dyskinesia (PCD). However, variants in *DNAH9* lead to nonsyndromic severe asthenozoospermia have yet to be reported.

**Methods:**

Whole exome sequencing (WES) was performed for two individuals with nonsyndromic severe asthenozoospermia from two non-consanguineous families, and Sanger sequencing was performed to verify the identified variants and parental origins. Sperm routine analysis, sperm vitality rate and sperm morphology analysis were performed according the WHO guidelines 2010 (5th edition). Transmission electron microscopy (TEM, TECNAI-10, 80 kV, Philips, Holland) was used to observe ultrastructures of sperm tail. Quantitative realtime-PCR and immunofluorescence staining were performed to detect the expression of DNAH9-mRNA and location of DNAH9-protein. Furthermore, assisted reproductive procedures were applied.

**Results:**

By WES and Sanger sequencing, compound heterozygous *DNAH9* (NM_001372.4) variants were identified in the two individuals with nonsyndromic severe asthenozoospermia (F1 II-1: c.302dupT, p.Leu101fs*47 / c.6956A > G, p.Asp2319Gly; F2 II-1: c.6294 T > A, p.Phe2098Leu / c.10571 T > A, p.Leu3524Gln). Progressive rates less than 1% with normal sperm morphology rates and normal vitality rates were found in both of the two subjects. No respiratory phenotypes, situs inversus or other malformations were found by detailed medical history, physical examination and lung CT scans etc. Moreover, the expression of DNAH9-mRNA was significantly decreased in sperm from F1 II-1. And expression of DNAH9 is lower in sperm tail by immunofluorescence staining in F1 II-1 compared with normal control. Notably, by intracytoplasmic sperm injection (ICSI), F1 II-1 and his partner successfully achieved clinical pregnancy.

**Conclusions:**

We identified *DNAH9* as a novel pathogenic gene for nonsyndromic severe asthenospermia, and ICSI can contribute to favorable pregnancy outcomes for these patients.

**Supplementary Information:**

The online version contains supplementary material available at 10.1186/s12958-021-00709-0.

## Background

Infertility is worldwide problem, affecting 8–15% couples of childbearing age, and male factors might contribute to nearly half of the cases [1–3]. Sufficient sperm motility is very important for sperm entering the female genital tract and oocyte fertilization [4]. According to the WHO guideline (2010), progressive motility < 32% or total motility < 40% in at least twice semen analysis at different times were defined as asthenospermia [5]. As one of the most common factors contributing to male infertility, asthenospermia might be associated with some cellular and molecular factors. Firstly, flagella/cilia, which is an important component of sperm tail structure, play a vital role in sperm motility system. Structural defects of flagella/cilia in sperm tail affect sperm motility directly. Secondly, ion channels, especially calcium channel is essential for sperm ion homeostasis, pH regulation and progressive motility. Thirdly, dysfunction of ATP production in mitochondria is also a principal feature of sperm immobility. Fourthly, some genetic mutations might have negative effects on sperm immobility. Fifthly, lifestyle risk factors, such as smoking, alcohol consumption, chemical pesticides exposure, etc. [6].

Severe asthenozoospermia, which is defined as the proportion of progressive spermatozoa less than 1%, is a tricky problem in infertile men [7, 8]. Previous studies have presented that genetic disorders might be related to severe asthenospermia. Sha et al. has found that *EIF4G1* (OMIM: 600495) was a candidate gene of severe asthenospermia in one patient, whose sperm was revealed with mitochondrial sheath defects [9]. Similarly, Xu et al. identified a homozygous *SPAG17* (OMIM: 616554) mutation was associated with severe asthenozoospermia in a familial twins [10]. Additionally, *AKAP3* (OMIM: 604689), *AKAP4* (OMIM: 300185), *DNAH1* (OMIM: 603332), *CATSPER2* (OMIM: 607249) and *NSUN7* (OMIM: 6171855), etc. were also revealed to be associated with severe asthenozoospermia [11–15].

Furthermore, severe asthenospermia is also revealed as one of the representative features of primary ciliary dyskinesia (PCD, OMIM: 244400), which includes a wide motile dysfunction of cilia in respiratory tract and sperm, etc. Most of the PCD cases are associated with genetic mutations, and some genes have been identified, such as *DNAH5* (OMIM: 603335)*, DNAH9* (OMIM: 603330) and *DNAH11* (OMIM: 603339), etc. [16–18]. *DNAH9* encodes a part of outer dynein arm (ODA) heavy chains components, and expressed on the entire length of the sperm tail [16]. Several researches have reported that mutations in *DNAH9* caused PCD [19, 20]. Fassad et al. conducted a study including 536 patients with PCD, and 4 cases from 3 families were found carrying *DNAH9* loss-of-function mutations. One of the four cases was presented with marked asthenospermia, and other 3 cases were not tested fertility due to too young age [19]. Loges et al. investigated 548 individuals with classical PCD symptoms or suspected PCD, and found 5 cases with mutations in *DNAH9.* However, no sperm-related examinations were performed in these cases [20].

Although mutations in *DNAH9* have been identified to be an important cause of syndromic asthenospermia, such as PCD, the association between *DNAH9* and nonsyndromic asthenospermia has not been examined. And whether *DNAH9* variants have effects on structures and ultrastructures of sperm tail, remain unclear. In this study, whole exome sequencing (WES) was performed in two Chinese men with severe nonsyndromic asthenospermia, and compound heterozygous variants in *DNAH9* were identified in these two cases.

## Methods

### Subjects

Two Han Chinese patients, presenting to the Reproductive Center of the First Affiliated Hospital of Anhui Medical University (Hefei, China) and the Xiamen Maternity and Child Care Hospital (Xiamen, China) respectively, and diagnosed with severe asthenospermia were enrolled in this study. Both subjects met several criteria as follows: without obvious PCD-related symptoms, such as chronic cough, recurrent infections of airways, as well as situs inversus and laterality disorders, etc.; with normal somatic karyotypes (46, XY); with normal semen volume (≥1.5 ml), sperm concentration (≥15 × 10^6^/ml), total sperm count (≥39 × 10^6^/ml); with a proportion of progressive spermatozoa < 1%; with a proportion of normal sperm morphology ≥4%; with normal sperm vitality rates (≥58%). Two subjects with normal fertility and normal semen characteristics were enrolled as control group. Peripheral whole blood of all cases was collected.

### Ethical approval

This study was approved by the Ethics Committee of the First Affiliated Hospital of Anhui Medical University and the Ethics Committee of Xiamen Maternity and Child Care Hospital. All the individuals and their family members, as well as two controls signed written informed consents after having received complete information about the research.

### Sperm analysis

Sperm routine analysis, sperm vitality rate and sperm morphology analysis were performed according the WHO guidelines 2010 (5th edition) [5].

### WES, bioinformatic analysis and sanger sequencing

DNA from whole peripheral blood was used to perform WES and bioinformatic analysis. And the protocols were in accordance with previous research [9, 21]. Sanger sequencing was performed to verify the identified variants and parental origins. The primers are listed in Supplementary Table 1.

### Transmission electron microscopy

Sperm sample was prepared and treated as described previously [9, 21]. Subsequently, transmission electron microscopy (TEM, TECNAI-10, 80 kV, Philips, Holland) was used to observe ultrastructures of sperm tail.

### Quantitative real-time PCR (QRT-PCR) and immunofluorescence staining

QRT-PCR experiment of DNAH9-mRNA and immunofluorescence staining experiment of DNAH9-protein in sperm were performed in accordance with previous study [9, 21]. The primary anti-DNAH9 was Anti-Dynein heavy chain antibody (ab133968, ABCAM). And the PCR primers were presented in Supplementary Table 2.

### Assisted reproductive procedures

The spouse of F1 II-1 underwent standard controlled ovarian hyperstimulation, and oocyte retrieval. Very few slightly motile spermatozoa were used for subsequent intracytoplasmic sperm injection (ICSI). The embryos were cultured to day 5 or day 6. Two viable thawed embryos were transferred 2 months later. Clinical pregnancy was confirmed by ultrasound performed 28 days after embryo transfer.

## Results

### Compound heterozygous variants in DNAH9 were identified in the individuals with severe asthenozoospermia

**Fig. 1:**
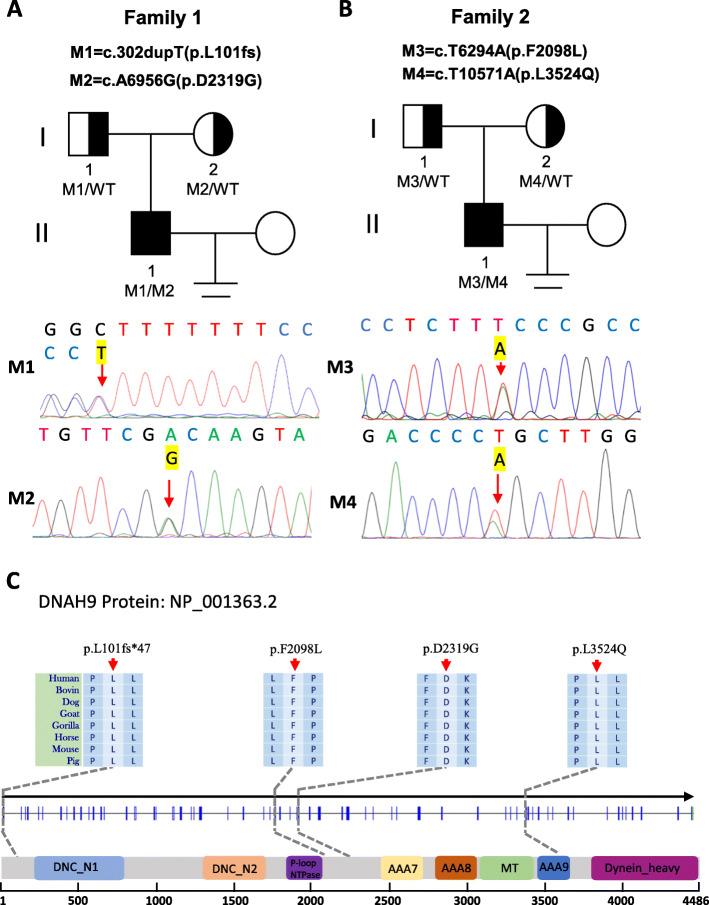
Variants of *DNAH9* in the two patients. **a-b** The two families affected by the variants in *DNAH9*. The red arrows indicate mutated positions in the Sanger sequencing results. **c** The mutated positions of *DNAH9* are conserved among species. And the red arrows indicate the locations of *DNAH9* variants occurred in the domains of DNAH9 protein. M, *DNAH9* variant; WT, wild type

To detect the cause of severe asthenozoospermia in these two patients, WES analysis was performed. Bioinformatical analysis were applied to seek for meaningful homozygous or compound heterozygous variants. Potentially pathogenic variants were obtained according to criteria as follows: allele frequencies < 1% in the ExAc_all, 1KGP and gnomAD databases; potentially high pathogenicity predicted by Sorting Intolerant From Tolerant (SIFT), PolyPhen-2, and Mutation Taster; extremely high expression in the testis. After the bioinformatical analysis, both of the two cases were found to carry compound heterozygous variants in *DNAH9*, the only gene associated with spermatozoa activity in genes met the above criteria. Subsequently, validation of Sanger sequencing for *DNAH9* was performed in the two cases and their parents. Compound heterozygous variants in *DNAH9* were validated in these two patients, and their parents were determined to be heterozygous carriers. The detailed information were presented in Table 1, Fig. 1 and Supplementary Fig. 1. The predicted part three-dimensional structure of mutated DNAH9 residues were presented in Supplementary Fig. 2 by SWISS-MODEL software (https://swissmodel.expasy.org/).

**Table 1:** Genetic information of *DNAH9* mutations of the two individuals

Individual	F1 II-1		F2 II-1	
**cDNA mutation**	c.302dupT	c.6956A > G	c.6294 T > A	c.10571 T > A
**Mutation type**	frameshift	nonsynonymous	nonsynonymous	nonsynonymous
**Protein alteration**	p.Leu101fs*47	p.Asp2319Gly	p.Phe2098Leu	p.Leu3524Gln
**Allele frequency in human population**				
**1KGP**	0	0	0	0
**ExAc_all**	0.0008	0.00002	0	0
**gnomAD**	0.0003	0.00002	0	0
**Functional prediction**				
**SIFT**	N/A	D	D	D
**PolyPhen-2**	N/A	D	D	D
**MutationTaster**	N/A	D	D	D

RefSeq accession number of *DNAH9* is NM_001372.4

Abbreviations: *1KGP* 1000 Genomes Project, *ExAc_all* all the data of Exome Aggregation Consortium, *gnomAD* the Genome Aggregation Database, *N/A* not applicable, *D* Disease-causing

### Sperm analysis and PCD-related symptoms of the two individuals carrying DNAH9 compound heterozygous variants

The clinical features and results of sperm tests were presented in Table 2. Both of the patients were found with proportions of progressive spermatozoa less than 1%, normal sperm vitality, and normal morphological sperm rates. No significant defects in sperm tail were found by Papanicolaou Staining method. Additionally, no PCD-related symptoms, such as chronic cough, recurrent infections of airways, as well as situs inversus and laterality disorders, etc., were found in these two cases. And the CT lung screening showed a normal result in the F1 II-1 (Supplementary Fig. 3). To further exclude possible ultrastructural defects, we performed TEM for spermatozoa from F1 II-1. No obvious defects were found in mitochondrial sheath and flagellum in F1 II-1 compared with normal control. The typical “9 + 2” microtubule structure were found in spermatozoa from both F1 II-1 and the control. Similarly, the typical structure of ODA was present in F1 II-1 and control (Supplementary Fig. 4).

**Table 2:** Clinical Features of the Two Individuals Carrying *DNAH9* Mutations

Individual	F1 II-1		F2 II-1	
**Age**	29		28	
**Semen analysis**	Sample 1	Sample 2	Sample 1	Sample 2
Semen volume (mL)	4.2	5.2	2.8	3.2
Sperm concentration (10^6^/mL)	133.7	55.3	78.6	43.8
Progressive motility (%)	**0.3**	**0.8**	**1.0**	**0.5**
Normal morphology (%)	6.5	5	4	4.5
Sperm vitality (%)	75	82	68	72
DFI (%)	10.18	N/A	N/A	N/A
**PCD-related phenomenon**				
Rhinosinusitis	No		No	
Wet cough	No		No	
Otitis media	No		No	
Bronchiectasis	No		No	
Situs inversus	No		No	
Congenital heart disease	No		No	

Abbreviations: *DFI* DNA fragmentation index, *N/A* Not applicable, *PCD* Primary ciliary dyskinesia

Bold characters indicate abnormal values

### Expression of DNAH9 was decreased in spermatozoa from F1 II-1

**Fig. 2:**
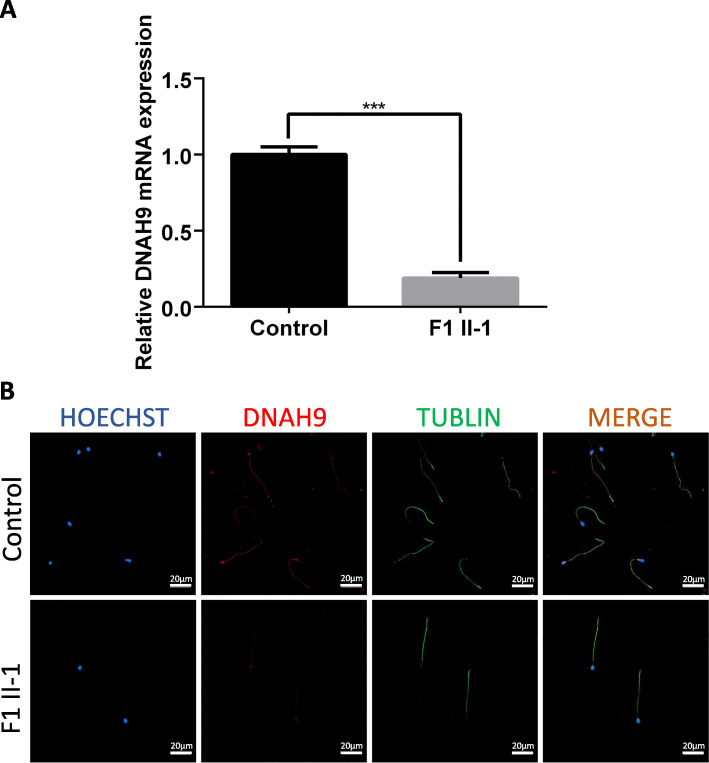
**a** The expression of DNAH9-mRNA in spermatozoon is significantly lower in F1 II-1 compared with normal control. **b** Immunofluorescence staining in spermatozoon from the *DNAH9*-mutated F1 II-1 and normal control. *DNAH9* immunostaining (red) was primarily concentrated at the sperm neck and flagellum in a punctate pattern, while the immunostaining was decreased in the sperm of F1 II-1. Hoechst (blue) was stained as a nuclear marker. The anti-tubulin (green) was stained as a marker of flagellum

As seminal fluid of F2 II-1 was not obtained, only spermatozoa from F1 II-1 were examined to determine the potential effect of variants on *DNAH9*. In the healthy control sperm, DNAH9 is highly expressed in sperm tail by immunofluorescence staining. However, expression of DNAH9 is lower in sperm of F1 II-1. In order to determine expressive level of mRNA of DNAH9, QRT-PCR was performed. And it was found that expressive level of DNAH9-mRNA in F1 II-1 was significantly lower than normal control (Fig. 2).

### Pregnancy outcome of patient with DNAH9 variants

**Fig. 3:**
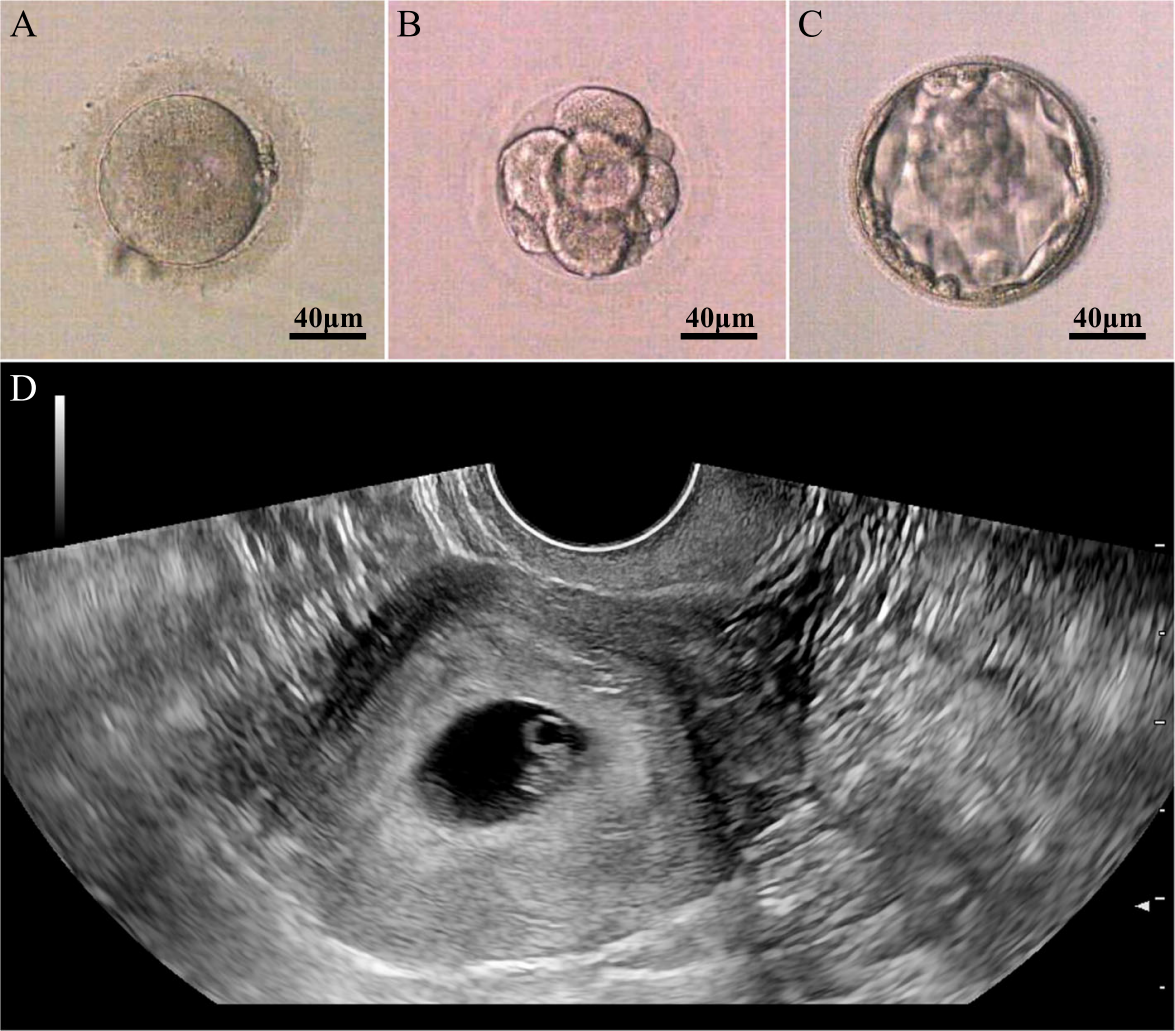
Typical morphology of the implanted embryo from F1 II-1. Well quality blastocysts formed after standard embryo culture and 2 embryos were transferred into the partner’s uterus. The embryo was implanted and yielded clinical pregnancy. **a**: 2 pronuclear (PN) fertilization, (**b**): 8-cell stage embryo, (**c**): blastocyst stage embryo.(**d**): the ultrasonography of gestational sac. Scale bar:40 μm

Standard controlled ovarian hyperstimulation, oocyte retrieval, and ICSI was performed for wife of F1 II-1. Sixteen oocytes in MII stage were retrieved. All the eggs got fertilization, and 11 fertilized eggs reached D5/D6 embryos. Subsequently, two high-quality D5 embryos were transferred after two-month frozen. The hCG level was 1300 mIU/ml in the blood 14 days after transfer, and clinical pregnancy was verified by ultrasound performed 28 days after embryo transfer (Fig. 3).

## Discussion

In this present study, we found two patients with severe asthenozoospermia harboring compound heterozygous variants in *DNAH9*. And the expression of DNAH9 was significant decreased in one patient with *DNAH9* variants, although no obvious morphological and ultrastructural defects were found. Moreover, no other PCD-related symptoms were found in these two cases. These results presented that *DNAH9* variant is a novel causative genetic etiology of nonsyndromic severe asthenozoospermia.

*DNAH9*, mapping to 17p12, is a human axonemal dynein beta heavy chain gene. There are 69 exons in this 14 kb-long cDNA of *DNAH9*, which encode a protein as a core component of outer dynein arms in cilia and flagella [22]. As cilia or flagella are highly conserved in varieties of human cells and organs, the mutations of *DNAH9* may be associated with multisystemic disorders, including bronchiectasis, recurrent respiratory tract infections, and male infertility, etc. [16, 19, 20, 23]. In the study conducted by Fassad et al., all the four patients carrying *DNAH9* mutations had syndromic clinical features, such as rhinosinusitis, wet cough and situs inversus, etc. [19]. Similarly, Loges et al. also found that respiratory phenotype or laterality defect in all the five individuals harboring *DNAH9* mutations. However, only one patient with *DNAH9* mutation was described with asthenospermia [20]. In our research, two cases carrying *DNAH9* compound heterozygous variants only presented with severe asthenospermia, without any respiratory symptoms or laterality defect by systemic medical history and physical examination. As no PCD-related symptoms in both cases and F2 II-1 losing follow-up, some invasive procedures, such as respiratory ciliary biopsy, etc. were not performed to test morphology or ultrastructure of other ciliary. Nonetheless, it was speculated that at least sperm motile defect was present in these patients with *DNAH9* compound heterozygous variants. The pathogenic *DNAH9* variants lead to significant decreased DNAH9 at mRNA and protein levels, however, we failed to observe obvious ultrastructure defects in sperm of F1 II-1. This could be due to the complexity of ODA, which composed of several heavy chain proteins, including DNAH5, DNAH8, DNAH11, DNAH17, et al. [19, 20, 24, 25]. These normal proteins can form a seemingly normal ODA structure, however, its dynamic ability damaged dramatically, causing severe asthenozoospermia. This in turn validated the essential role of DNAH9 in ODA function.

The diverse types of the variants in *DNAH9* might lead to different disease phenotypes and varying ciliary defects. The clinical phenotype in these two patients are less severe compared with other individuals harboring *DNAH9* mutations with classical PCD symptoms. The identified compound heterozygous variants in these two cases both includes missense variants, milder genetic defects, which might have only mild harmful effects on structure and function of DNAH9. Therefore, only severe sperm motility defects were presented in these two cases. It was partially supported by the normal morphological and ultrastructure in sperm of F1 II-1. Unlike cases in the studies of Fassad and Loges et al., no laterality defects were found in these two patients. DNAH9 is an important core of ODA, whose mutations may result in impaired ciliary motility and subsequent impaired or disturbed leftward fluid flow within the embryonic node. Therefore, only nearly half of patients with PCD present situs inversus. Additionally, the milder genetic defects of *DNAH9* in these two cases may had little effects on the fluid flow within the embryonic node. These factors may contribute to the normal laterality in these two patients. Despite of *DNAH9,* diverse variants of some other candidate genes of PCD could also lead to various phenotypes. *DNAH1*, a core component of inner-arm heavy chain dynein, was also considered as a candidate gene causing PCD [26, 27]. Similarly, twelve patients with *DNAH1* variants were only exhibited multiple morphologic abnormalities of the flagella (MMAF) phenotype, but without PCD in the study conducted by Sha et al. [28].

Severe asthenozoospermia is a rare disease, and assisted reproduction techniques (ART), especially ICSI is an important and even sole method for these patients to get pregnant. The wife of F1 II-1 also received the examination of *DNAH9* gene, and no abnormal variants were found. Considering an autosomal recessive pattern of inheritance, the F1 II-1 couple underwent standard ICSI procedure, and get good clinical outcome. It is also the first time to report that ICSI can be recommended for severe asthenozoospermia caused by *DNAH9* variants.

Some limitations should also be taken into consideration. Firstly, the sperm sample of F2 II-1 can not be obtained. So all the experiments of sperm were only performed in F1 II-1. Additionally, respiratory ciliary was not collected for further experiments as a invasive procedure. Secondly, as the not enough sperm sample, western blot experiment was not performed to examine the protein expressive level of DNAH9 in F1 II-1. Thirdly, we did not explore the prevalence of *DNAH9* variant in large-scale population with nonsyndromic severe asthenospermia, which will be an important work in future.

## Conclusions

In summary, variants of *DNAH9* are novel pathogenic factors for not only syndromic severe asthenospermia, such as PCD, but also nonsyndromic severe asthenospermia. ICSI can be recommended as a first-line treatment for favorable pregnancy outcomes for these patients.

## Supplementary Information

**Additional file 1: Supplementary Table 1**. Primers used for verification of *DNAH9* mutations.

**Additional file 2: Supplementary Table 2**. Primers used for QRT-PCR assay of DNAH9 and β-actin.

**Additional file 3: Supplementary Fig. 1.** Sanger sequencing results of of two cases and their parents.

**Additional file 4: Supplementary Fig. 2.** The predicted part three-dimensional structure of mutated DNAH9 residues by SWISS-MODEL software (https://swissmodel.expasy.org/); WT, wild type.

**Additional file 5: Supplementary Fig. 3.** Diagnostic imaging tests excluded typical PCD signs. (A): The chest X rays showed the heart on the left. (B): The chest CT showed normal pulmonary bronchus. (C): The upper abdomen CT showed regular visceral structure.

**Additional file 6: Supplementary Fig. 4.** Sperm morphology and ultrastructure in the F1 II-1 with *DNAH9* compound heterozygous variants. (A-D) Normal spermatozoon from a healthy control man with normal fertility. (E-H) Spermatozoon from F1 II-1 with severe asthenospermia. Sperm morphology analysis showed normal long flagella in the control man (A), and F1 II-1 (E). TEM showed the typical “9 + 2” microtubule structure as well as normal outer dynein arms in spermatozoa of the control man (B-D), and F1 II-1 (F-H). Scale bars: 10 μm in (A) and (E); 1um in (B-D) and (F-H). CP, central pair of microtubules; PM, peripheral microtubule doublets; ODF, outer dense fiber; MS, mitochondrial sheath; TEM, transmission electron microscopy.

## Data Availability

The datasets used and/or analysed during the current study are available from the corresponding author on reasonable request.

## Abbreviations

PCD: Primary ciliary dyskinesia
WES: Whole exome sequencing
TEM: Transmission electron microscopy
ICSI: Intracytoplasmic sperm injection
ODA: Outer dynein arm
QRT-PCR: Quantitative real-time PCR
SIFT: Sorting Intolerant From Tolerant
MMAF: Multiple morphologic abnormalities of the flagella
ART: Assisted reproduction techniques
