# Supplementary material for: Novel variants in DNAH9 lead to nonsyndromic severe asthenozoospermia
Source: Reprod Biol Endocrinol. 2021 Feb 20;19:27. doi: 10.1186/s12958-021-00709-0 (PMC7896388; doi:10.1186/s12958-021-00709-0)
Supplement: Supplementary file 1 — Additional file 1: Supplementary Table 1. Primers used for verification of DNAH9 mutations. [file 12958_2021_709_MOESM1_ESM.docx]

**Supplementary table 1. Primers used for verification of *DNAH9* mutations.**

|  | **Primer Names** | **Primer Sequences (5'-3')** |
| --- | --- | --- |
| **F1 II-**1 | M1-F | CAGGGGCCTGGCAATACG |
|  | M1-R | TAACCCACCCTCACCTCCG |
|  | M2-F | GGCAGACTTGGGATGGAACC |
|  | M2-R | GGACAGGACAGAACATCAGCA |
| **F2 II-1** | M3-F | TAACACTGCCCACAGGATCA |
|  | M3-R | TGTGGGTCCCCAGTTAAGAG |
|  | M4-F | CAACTCCATAGCTTGCCACA |
|  | M4-R | ATGTGCTGCAGGGAGCTACT |
