# Supplementary material for: Novel variants in DNAH9 lead to nonsyndromic severe asthenozoospermia
Source: Reprod Biol Endocrinol. 2021 Feb 20;19:27. doi: 10.1186/s12958-021-00709-0 (PMC7896388; doi:10.1186/s12958-021-00709-0)
Supplement: Supplementary file 2 — Additional file 2: Supplementary Table 2. Primers used for QRT-PCR assay of DNAH9 and β-actin. [file 12958_2021_709_MOESM2_ESM.docx]

| **Supplementary table 2. Primers used for QRT-PCR assay of DNAH9 and β-actin.** | | | |
| --- | --- | --- | --- |
|  | **Primer Names** | **Primer Sequences (5'-3')** | **Tm** |
| ***DNAH9*** | Forward | GGATGACCTATGCTTTGCGAG | 60.8 |
|  | Reverse | CTGGTCCCGATTCTTCAAATGA | 60 |
| ***β-actin*** | Forward | CATGTACGTTGCTATCCAGGC | 60.8 |
|  | Reverse | CTCCTTAATGTCACGCACGAT | 60.2 |
