# Supplementary figures and images for: Novel variants in DNAH9 lead to nonsyndromic severe asthenozoospermia
Source: Reprod Biol Endocrinol. 2021 Feb 20;19:27. doi: 10.1186/s12958-021-00709-0 (PMC7896388; doi:10.1186/s12958-021-00709-0)

**A****Family 1****M1=c.302dupT(p.L101fs\*47)****M2=c.6956A>G(p.D2319G)**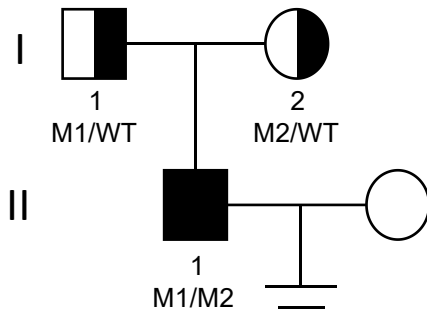**F1 II-1**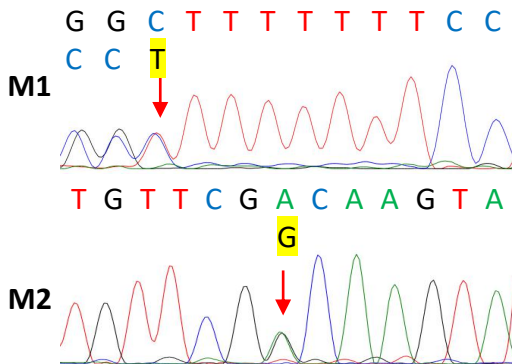**F1 I-1**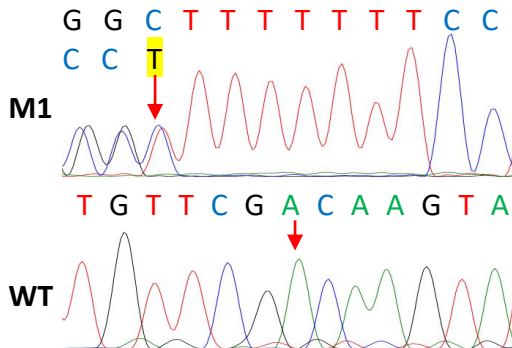**F1 I-2**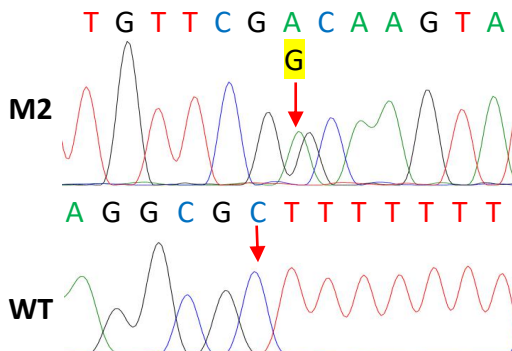**B****Family 2****M3=c.6294T>A(p.F2098L)****M4=c.10571T>A(p.L3524Q)**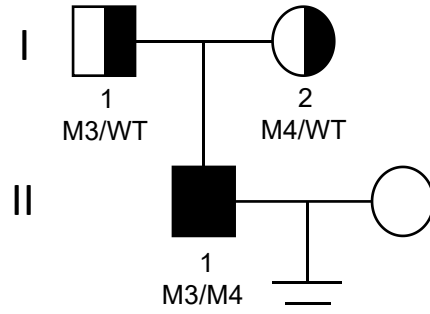**F2 II-1**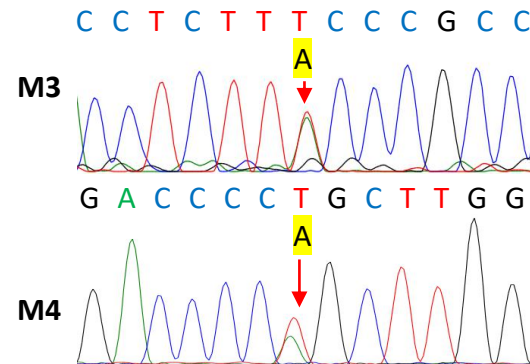**F2 I-1**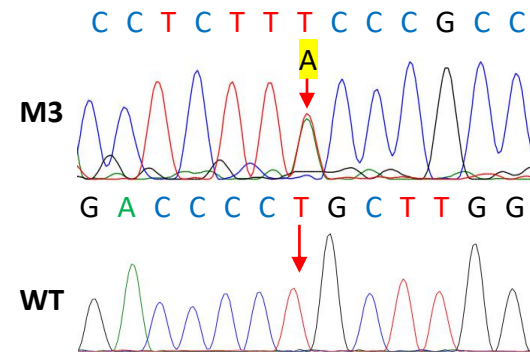**F2 I-2**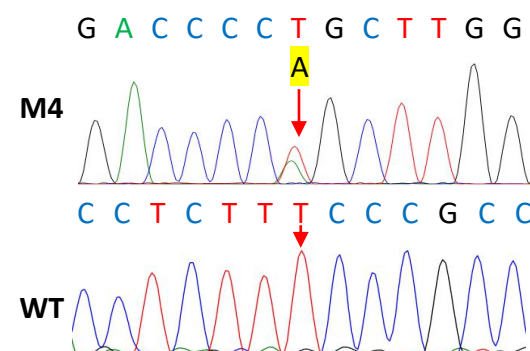

Supplement: Supplementary file 3 — Additional file 3: Supplementary Fig. 1. Sanger sequencing results of of two cases and their parents. [file 12958_2021_709_MOESM3_ESM.pdf]

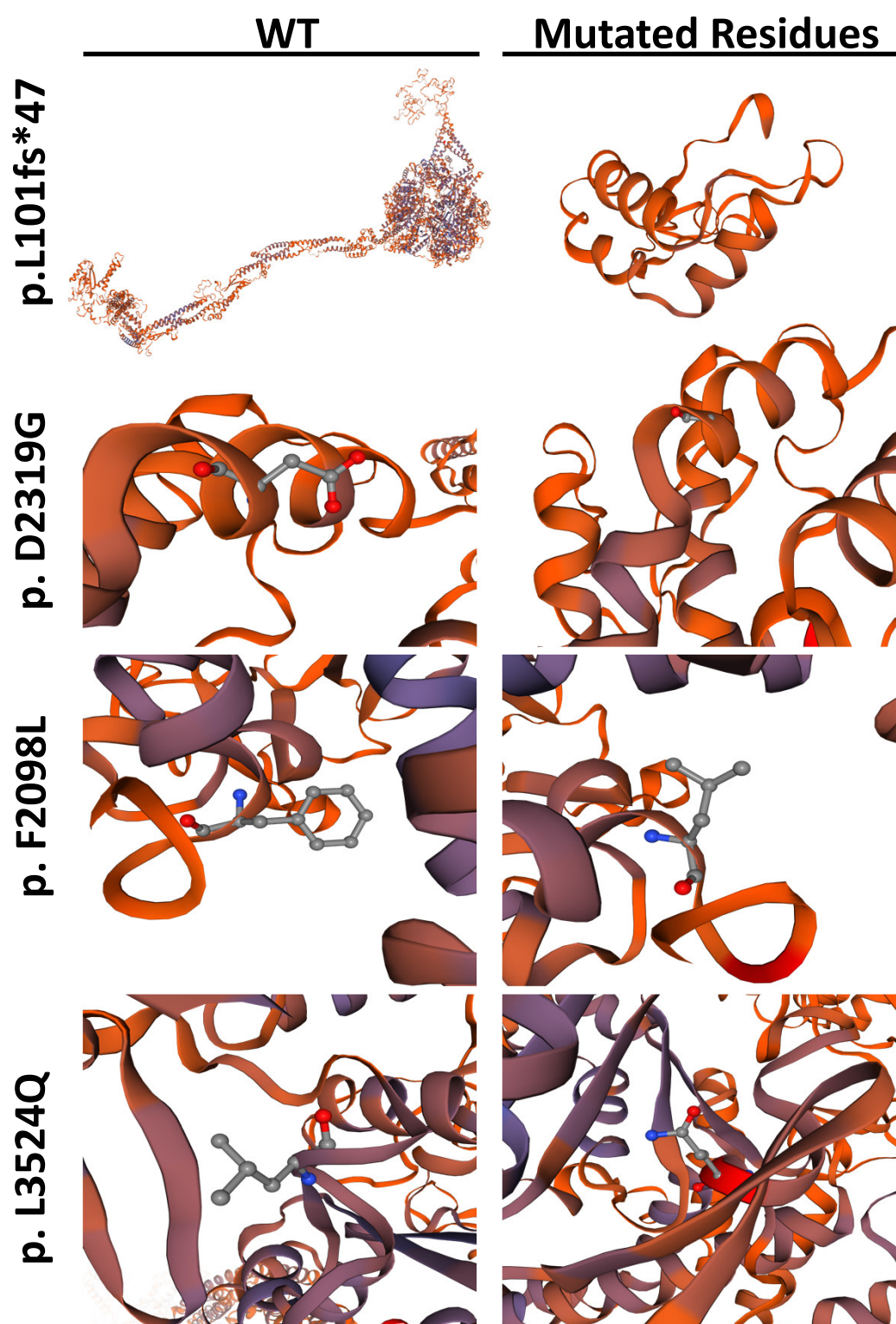

Supplement: Supplementary file 4 — Additional file 4: Supplementary Fig. 2. The predicted part three-dimensional structure of mutated DNAH9 residues by SWISS-MODEL software (https://swissmodel.expasy.org/); WT, wild type. [file 12958_2021_709_MOESM4_ESM.pdf]

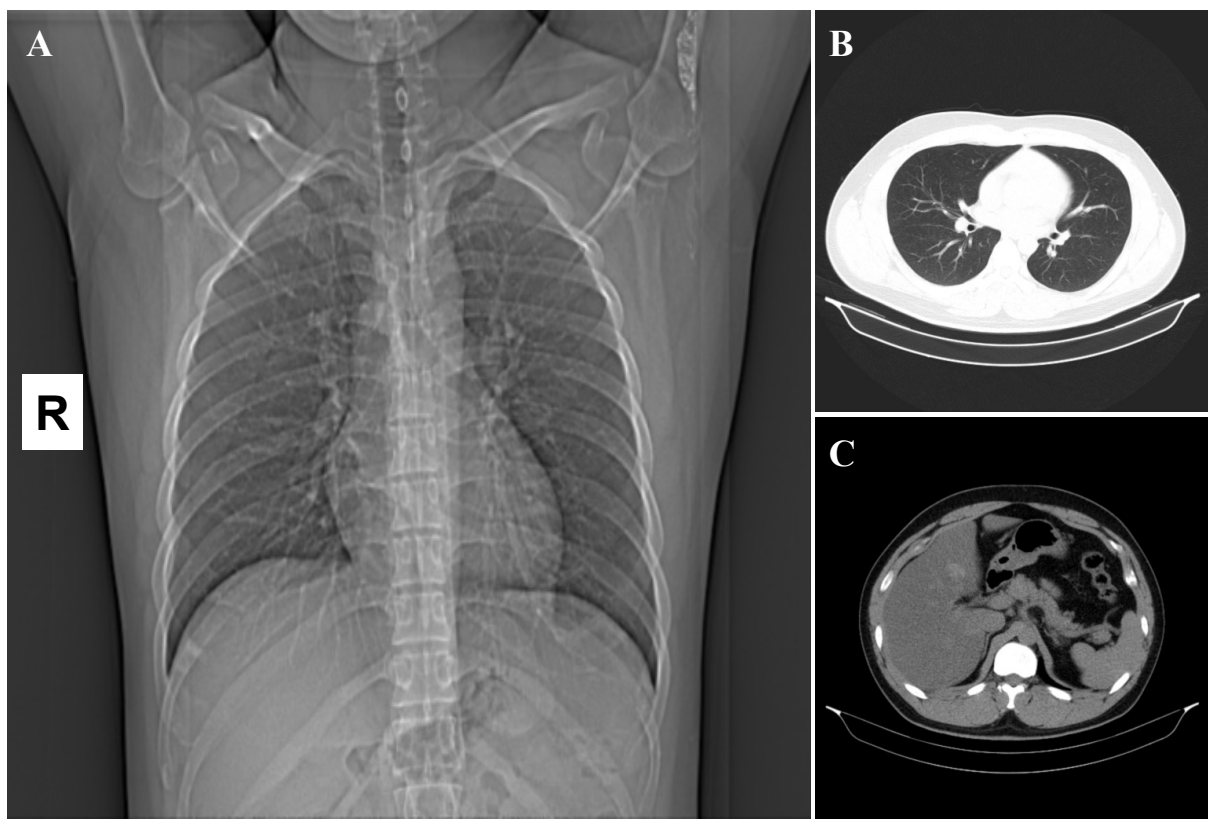

Supplement: Supplementary file 5 — Additional file 5: Supplementary Fig. 3. Diagnostic imaging tests excluded typical PCD signs. (A): The chest X rays showed the heart on the left. (B): The chest CT showed normal pulmonary bronchus. (C): The upper abdomen CT showed regular visceral structure. [file 12958_2021_709_MOESM5_ESM.pdf]

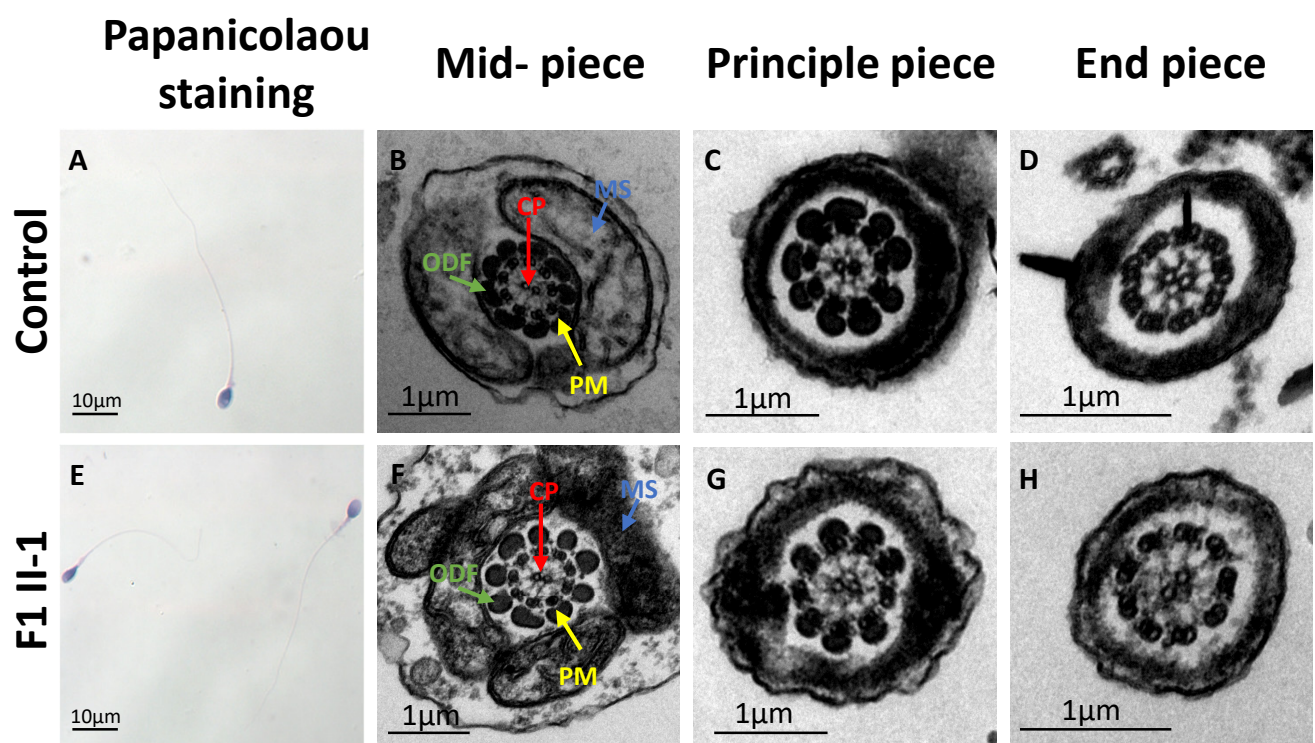

Supplement: Supplementary file 6 — Additional file 6: Supplementary Fig. 4. Sperm morphology and ultrastructure in the F1 II-1 with DNAH9 compound heterozygous variants. (A-D) Normal spermatozoon from a healthy control man with normal fertility. (E-H) Spermatozoon from F1 II-1 with severe asthenospermia. Sperm morphology analysis showed normal long flagella in the control man (A), and F1 II-1 (E). TEM showed the typical “9 + 2” microtubule structure as well as normal outer dynein arms in spermatozoa of the control man (B-D), and F1 II-1 (F-H). Scale bars: 10 μm in (A) and (E); 1um in (B-D) and (F-H). CP, central pair of microtubules; PM, peripheral microtubule doublets; ODF, outer dense fiber; MS, mitochondrial sheath; TEM, transmission electron microscopy. [file 12958_2021_709_MOESM6_ESM.pdf]
